# Spermiogenesis alterations in the absence of CTCF revealed by single cell RNA sequencing

**DOI:** 10.3389/fcell.2023.1119514

**Published:** 2023-03-30

**Authors:** Ulises Torres-Flores, Fernanda Díaz-Espinosa, Tayde López-Santaella, Rosa Rebollar-Vega, Aarón Vázquez-Jiménez, Ian J. Taylor, Rosario Ortiz-Hernández, Olga M. Echeverría, Gerardo H. Vázquez-Nin, María Concepción Gutierrez-Ruiz, Inti Alberto De la Rosa-Velázquez, Osbaldo Resendis-Antonio, Abrahan Hernández-Hernandez

**Affiliations:** ^1^ Graduate Program in Experimental Biology, DCBS, Universidad Autónoma Metropolitana, Unidad Iztapalapa, México City, Mexico; ^2^ Biología de Células Individuales (BIOCELIN), Laboratorio de Investigación en Patología Experimental, Hospital Infantíl de México Federico Gómez, México City, Mexico; ^3^ Coordinación de la Investigación Científica-Red de Apoyo a la Investigación, Universidad Nacional Autónoma de México e Instituto Nacional de Ciencias Médicas y Nutrición Salvador Zubirán, México City, Mexico; ^4^ Coordinación de la Investigación Científica-Red de Apoyo a la Investigación-Centro de Ciencias de la Complejidad, Universidad Nacional Autónoma de México, México City, Mexico; ^5^ Human Systems Biology Laboratory, Instituto Nacional de Medicina Genómica, Mexico City, Mexico; ^6^ BD Life Sciences Informatics, Ashland, OR, United States; ^7^ Laboratorio de Microscopía Electrónica, Facultad de Ciencias, Universidad Nacional Autónoma de México, Mexico City, Mexico; ^8^ Laboratorio de Fisiología Celular y Medicina Traslacional, Departamento de Ciencias de la Salud, Universidad Autónoma Metropolitana-I, Mexico City, Mexico

**Keywords:** ScRNA-seq, mouse testis, spermatogenesis, CTCF, sperm

## Abstract

CTCF is an architectonic protein that organizes the genome inside the nucleus in almost all eukaryotic cells. There is evidence that CTCF plays a critical role during spermatogenesis as its depletion produces abnormal sperm and infertility. However, defects produced by its depletion throughout spermatogenesis have not been fully characterized. In this work, we performed single cell RNA sequencing in spermatogenic cells with and without CTCF. We uncovered defects in transcriptional programs that explain the severity of the damage in the produced sperm. In the early stages of spermatogenesis, transcriptional alterations are mild. As germ cells go through the specialization stage or spermiogenesis, transcriptional profiles become more altered. We found morphology defects in spermatids that support the alterations in their transcriptional profiles. Altogether, our study sheds light on the contribution of CTCF to the phenotype of male gametes and provides a fundamental description of its role at different stages of spermiogenesis.

## Introduction

The CCCTC-binding factor (CTCF) is a zinc finger (ZF) protein with highly conserved DNA binding activity that has been suggested to play a crucial role in the evolution of Metazoa (Heger et al., 2012). This protein has three main domains: the N-terminal domain responsible to engage the SA2-SCC1 cohesin subcomplex (Li et al., 2020), the central domain with 11 zinc fingers, from which ZF three to seven bind DNA, while the remaining ZF modulate the stability of CTCF binding by interacting with adjacent DNA modules (Arzate-Mejía et al., 2018) and the C-terminal domain that interacts with RNAPII, RNA, Kaiso and TFII-I (Arzate-Mejía et al., 2018). CTCF throughout interaction with its partners at several genome positions organizes the genome in space and regulates gene expression in a time- and cell-specific manner (Sun et al., 2022). Thus, CTCF is recognized as a genome architectonical protein with a pivotal role in a wide variety of biological processes. Functional studies in early development of vertebrates showed that its absence induces embryo lethality (Delgado-Olguín et al., 2011; Moore et al., 2012; Arzate-Mejía et al., 2018). Additionally, loss of CTCF causes premature neurogenesis, leading to depletion of the progenitor pool and a microcephaly phenotype at birth (Watson et al., 2014). Conditional deletion of CTCF from cardiac progenitors causes severe cardiac defects and death on embryonic day 12.5 (Gomez-Velazquez et al., 2017). CTCF is also involved in retinal cell differentiation, as when it is overexpressed in mouse embryos it results in underdeveloped eyes, small lenses, and reduced populations of cells in the retina (Li et al., 2004). During gametogenesis, CTCF has also been suggested to play an important role as its depletion from mouse oocytes, causes changes in gene expression and mild meiotic defects resulting in embryonic lethality (Wan et al., 2008). In mouse spermatocytes with conditional deletion of *Ctcf,* altered sperm count production and infertility have been reported (Hernández-Hernández et al., 2016).

Sperm production in mammals or spermatogenesis involves complex processes of cell division and specialization that produce highly specialized germ cells whose only purpose is to fertilize female germ cells and produce new organisms (White-Cooper and Bausek, 2010). In male gonads, within the seminiferous tubules, the diploid spermatogonia cells undergo mitosis to ensure a constant supply of immature germ cells. A specific type of spermatogonia commits to the meiotic process and its DNA is duplicated prior to genetic exchange between homologous chromosomes. After two uninterrupted rounds of meiotic cell divisions, four haploid cells with recombination events are produced (Garg and Martin, 2016). These haploid cells or spermatids engage in a differentiation process that prepares them for their final journey: the pursuit of a female germ cell. During this process known as spermiogenesis, there are changes in cell morphology that produce the classic torpedo-like shape of mature sperm (Dadoune, 2003; Torres-Flores and Hernández-Hernández, 2020).

During all steps of spermatogenesis, chromatin structure and transcriptional profiles are very dynamic and specific (Jung et al., 2017; Green et al., 2018; Hermann et al., 2018; Luo et al., 2020). Recent studies of chromatin organization in meiotic and haploid stages have shown that the three-dimensional organization of the genome is rather different in these two stages. At the meiotic stage, the genome is organized in meiosis-specific topologically associating domains (TADs) (Luo et al., 2020), whereas in haploid cells, it is completely reorganized giving rise to the sperm specific epigenome, which is necessary to recapitulate chromatin structure during embryo development (van de Werken et al., 2014; Jung et al., 2017; Luo et al., 2020). Proper histone retention in mammal sperm has been shown to play a role in inter- and intra-transgenerational epigenetic inheritance (Siklenka et al., 2015). Although substantial progress about the interplay among chromatin organization, establishment of sperm epigenome and maintenance of epigenetic information in the sperm has been made, only few proteins have been associated to the establishment of the sperm epigenome. In this regard, it has been suggested that genome architectural proteins like CTCF and cohesin complexes plus specific epigenetic marks in retained histones contribute to the establishment of the sperm epigenome in mice (Jung et al., 2019). Furthermore, CTCF has been involved in the processes of histone retention and the proper formation of mature spermatozoa (Hernández-Hernández et al., 2016). However, the impact of CTCF during each step of spermiogenesis has been difficult to analyze. Previously, we generated a conditional Knock-out mouse (*Ctcf*-cKO) in which *Ctcf* is deleted at the beginning of the meiotic stage. In these *Ctcf*-cKO mice, meiosis and spermiogenesis are completed, albeit displaying low counts of sperm with defects in morphology and histone retention to some extent. Unfortunately, we were not able to identify the transcriptomic alterations produced at each stage of spermatogenesis due to the high heterogeneity of cell types in the testis (Hernández-Hernández et al., 2016).

In this work, by single-cell RNA sequencing (scRNA-seq), we have identified the transcriptomic defects produced by the absence of CTCF in the main stages of spermatogenesis. We found that transcriptional alterations are mild at the early stages of spermatogenesis. However, in late stages we observed significant alterations in transcriptional profiles, biological functions, and morphologic defects. In general, we characterized the impact of CTCF in several stages of spermatogenesis.

## Materials and methods

### Animals

Animal care was in accordance with the regulations established by the “Ethic and Scientific Responsibility Committee” of the Faculty of Sciences, UNAM (PI_2019_08_002) and by the “Ethic Committee” of the Hospital Infantil del Mexico Federico Gomez (HIM/2021/061). We have previously generated and validated a transgenic mouse strain that undergoes depletion of CTCF during spermatogenesis (Hernández-Hernández et al., 2016). For this work, we used male mice of 12–15 weeks old to minimize age-related variations. We compared *Ctcf* conditional knockout mice (*Ctcf*-cKO, with a *Stra8-iCre-Ctcf* ^f/Δ^ genotype) *versus* wild type (WT) littermates with genotypes *Ctcf* ^f/f^, *Ctcf* ^wt/f^ or *Ctcf* ^wt/wt^.

### Single cell RNA sequencing

We performed spermatogenic cell dissociation from testes as previously reported (Kossack et al., 2013) with some modifications. Briefly, we extracted and removed the tunica albuginea from the testes of two WT and two *Ctcf*-cKO mice (number of biological replicates that have previously been used for this kind of studies, (Hermann et al., 2018; Lukassen et al., 2018)). We gently spread seminiferous tubules in a small Petri dish and transferred them to a 15 ml conic tube containing 5 ml of enzymatic buffer (20 mM Hepes pH 7.2, 6.6 mM sodium pyruvate, 0.05% lactate in 1x HBSS), supplemented with 100 U/ml collagenase type I, 2 U/ml DNase. The tubules were incubated for 25 min at 32°C and the suspension was filtrated through a 40 μm nylon mesh. FBS was added to the filtrated solution to a final concentration of 2%. The filtrates of two tubes from the *Ctcf-*cKO mouse were pooled in a new tube. The cells were then pelleted by centrifugation at 1,000 *g* for 10 min at 4°C, carefully aspirated the supernatant and added 1 ml of HBSS enzyme digestion buffer. We then thoroughly mixed and transferred the suspension to a 1.5 ml tube. Cells were pelleted by centrifugation at 1,000 *g* for 10 min at 4°C and the supernatant was aspirated.

To remove as many erythrocytes as possible from the cell suspension, we washed the pellet three times with ice cold PBS. Finally, we pelleted the cells by centrifugation at 1,000 *g* for 10 min at 4°C and removed the supernatant. Spermatogenic cells were resuspended in PBS plus 0.2% BSA and cell concentration was adjusted to 2.5 × 10^6^ cells/ml. Only cell suspensions with viabilities greater than 90% were used for downstream analysis. We performed single cell isolation and cell barcoding with the Droplet-based Single Cell Isolator (one-touch ddSEQ, Bio-Rad) according to the manufacturer’s instructions. Subsequently, we prepared single cell barcoded RNA-Seq libraries using Nextera technology included in the SureCell WTA 3′Library Prep Kit (Illumina). Next-generation sequencing was performed in a NextSeq (Illumina) with an attainable depth of 250,000 reads/cell. Four datasets from two different WT and two different *Ctcf*-cKO mice (two biological replicates per group as commonly reported for single cell RNA-seq studies in mice (Hermann et al., 2018; Lukassen et al., 2018)) were generated and used for further analysis.

### Data analysis

To process single cell mRNA sequencing data, we generated the count expression matrixes by aligning the raw data to the mouse genome (GRCm38.98) following previous reports using the default parameters (Romagnoli et al., 2018; Tian et al., 2018). For the downstream analysis of the count expression matrices, we used Harmony 0.1.0 package (Korsunsky et al., 2018) for batch correction and Seurat 4.1.0 package (Satija et al., 2015) for dimensional reduction and clustering. Finally we used Monocle3 1.2.6 package for pseudotime analysis (Trapnell et al., 2014). Briefly, we created a Seurat object with the count matrix and performed a standard preprocessing workflow. We obtained a count matrix with 406 and 503 cell barcodes for WT and *Ctcf*-cKO, respectively. We then used Seurat to perform quality control metrics and to remove cells that have unique feature counts greater than 7,000 or less than 200, and cells with more than 10% mitochondrial genes, as commonly reported elsewhere (Bailur et al., 2019; Okada et al., 2022). After this we obtained a count matrix with 405 and 503 cell barcodes for WT and *Ctcf*-cKO, respectively (Supplementary Figure S1A).

Data normalization, scaling using the “LogNormalize”, “ScaleData” and, feature selection “vst” methods were performed before batch correction with Harmony. Finally, dimensionality reduction and cell clustering were performed with Seurat (according to standard tutorials https://satijalab.org/seurat/articles/pbmc3k_tutorial.html) using PCA dimension: 1:15 and cluster resolution: 0.9. For differential gene expression, we use default “Fold.change” parameters inside Seurat. The similarity heatmap between our clusters and previously validated clusters (Hermann et al., 2018) was performed by comparing the significantly expressed cluster-specific markers in both studies (using the function “FindAllMarkers” in Seurat and selecting genes with a *p*-value adjusted <0.05) and with the formula:

$$\frac{\left(Genes\;in\;common\right)x100}{\begin{array}{c} \left[\left(N1\;genes+N2\;genes\right)-Genes\;in\;common\right]\\ \;\\ \; \end{array}}$$ (1)

Where:

Genes in common: are the number of genes shared by two clusters.

N1 genes: total number of significantly expressed genes in reference cluster.

N2 genes: total number of significantly expressed genes in the experimental cluster.

### Pathway enrichment analysis

To analyze functional annotation of the up and downregulated genes (*p*-value adjusted <0.05), we used DAVID v6.8 (The Database for Annotation, Visualization, and Integrated Discovery) (Huang et al., 2009a), using the whole genome of *Mus musculus* as background for enrichment analysis. For the pathway enrichment analysis, we used the GSEA computational method (Gene Set Enrichment Analysis) (Subramanian et al., 2005) available at http://software.broadinstitute.org/gsea/). We obtained and used all annotations to the gene set from the GSKB database (Lai et al., 2016). Statistical significance was assigned with an FDR <0.05 and a *p*-value <0.01.

### Enrichment maps

We constructed enrichment maps with the pathway enrichment analysis information in Cytoscape 3.9.1 software. Using a Jaccard index values ≥0.4 we associated statistically significant enriched pathways according to their shared genes. We set the biological function collections tags with the AutoAnnote app. A preliminary map was constructed in Cytoscape, and then the final design was performed using Affinity Designer 1.10.4.

### Histological sections processing

We performed immunofluorescent and electron microscopy as previously reported (Hernández-Hernández et al., 2016). We employed the next primary antibodies: rabbit polyclonal anti-Transition Protein 1 (TNP1) (Abcam, ab73135); mouse monoclonal antibody anti-Transition Protein 2 (TNP2) (Santa Cruz Biotechnology, sc-393843); mice anti-Protamine 1 (PRM1) and mice anti-Protamine 2 (PRM2) (Briar Patch Biosciences LLC, Hup1N and Hup2B, respectively). For immunofluorescent microscopy, we use the secondary antibodies: Goat-anti-rabbit IgG, Alexa Flour 594 (Invitrogen, A-11008); Goat-anti-mouse IgG, Alexa Flour 488 (Invitrogen, A28175) and Goat-anti-mouse IgG, Alexa Flour 594 (Invitrogen, A32742).

## Results

### Single cell RNA sequencing reveals the populations of spermatogenetic cells in WT and *Ctcf-*cKO mice

**FIGURE 1:**
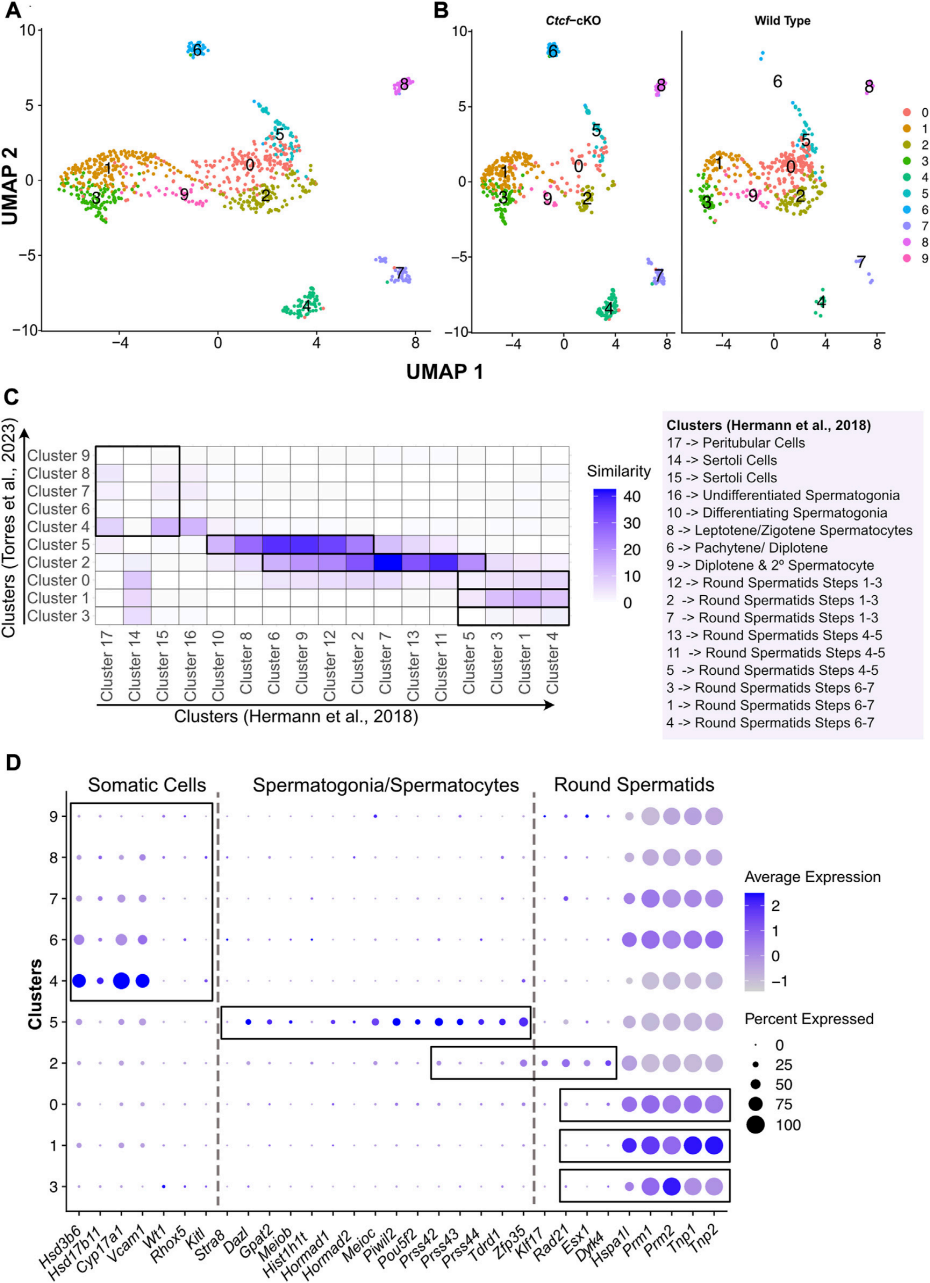
Clustering, validation, and identification of spermatogenesis populations from WT and *Ctcf-*cKO testes. **(A)** UMAP visualization of 10 cell clusters from WT and *Ctcf*-cKO cell populations. **(B)** Visualization of cell clusters by genotype. Clusters are distinguished by color according to the key color. **(C)** Heatmap displaying the percentage of similarity shared between our 10 clusters *versus* 17 clusters obtained in Hermann, et al., 2018 study. We considered a *p*-value adjusted <0.05 in genes list from each cluster. Cluster identity is mentioned to the right of the heatmap. **(D)** Dot plot of the proportion of cells in the respective cluster expressing each marker (dot size), and the average expression (color scale).

We previously reported that conditional deletion of *Ctcf* during mouse spermatogenesis produces abnormal mature sperm (Hernández-Hernández et al., 2016). To elucidate the major stages of spermatogenesis at which these abnormalities take place, we performed scRNA-seq from isolated spermatogenic cells from two WT and two *Ctcf-*cKO mice. We performed quality controls to filter out cells with high/low gene counts and high percentages of mitochondrial genes (Supplementary Figure S1A), followed by batch correction to eliminate technical biases and to corroborate significative integration of the different datasets. Subsequently, we performed unsupervised clustering projected onto uniform manifold approximation and projection for dimension reduction (UMAP). The graph-based clustering revealed 10 clusters in both genotypes (Figures 1A, B). Although both mice displayed the same clusters, we found changes in the proportions of cells in opposite genotypes within the same cluster (Supplementary Figures S1B, S1C).

Since we used a droplet-based platform with a cell throughput lower than the most widely used platform (ddSeq-BioRad *versus* Chromium from 10X Genomics, respectively), we decided to increase the number of reads per cell to estimate the true transcriptional state of cells in our system (Zhang et al., 2020). We first addressed whether our 10 clusters with 405 and 503 cells from the WT and *Ctcf-*cKO testes, respectively; represented the entire spermatogenetic process. Therefore, we compared and plotted the percentage of similarity (defined as the percentage of shared markers between two clusters) between the differentially expressed genes (DGE) of each of our 10 clusters and the DGE of each of the 17 clusters obtained elsewhere by performing a scRNA-seq of 4,651 cells from two different WT mice (Hermann et al., 2018). We observed that clusters 4, 7, 8 of our analysis display the highest similarity to clusters containing Sertoli and Peritubular cells (clusters 15 and 17 in (17)) (Figure 1C). Cluster 5 has transcriptional profiles related to spermatogonia/spermatocytes (clusters 10, 8, 6 and 9 in (17)), cluster 2 is similar to spermatocytes/round spermatids (clusters 6, 9, 12, 2, 7, 13, 11 and 5 in (17)). Finally, clusters 0, 1 and 3 showed similarity to early, mid, and late-round spermatids (clusters 12, 2, 7, 13, 11, 5, 3, 1 and 4 in (17)) (Figure 1C).

To support previous associations and assign identity to our clusters, we inspected the expression of specific markers for Leydig, Sertoli, spermatogonia, meiotic and round spermatid cells previously reported in two different scRNA-seq studies (Hermann et al., 2018; Lukassen et al., 2018). Consequently, we found that clusters 4, 6, 7, 8 and 9 display markers for somatic cells (Sertoli and Leydig cells) (Figure 1D), while the cells in cluster 5 express markers for spermatogonia and meiotic cells (spermatocytes) (Figure 1D). Cluster 2 shows expression of markers for spermatocytes and round spermatids cells (Figure 1D). Finally, we noticed that clusters 3, 1 and 0 display markers for round spermatids (Figure 1D).

**FIGURE 2:**
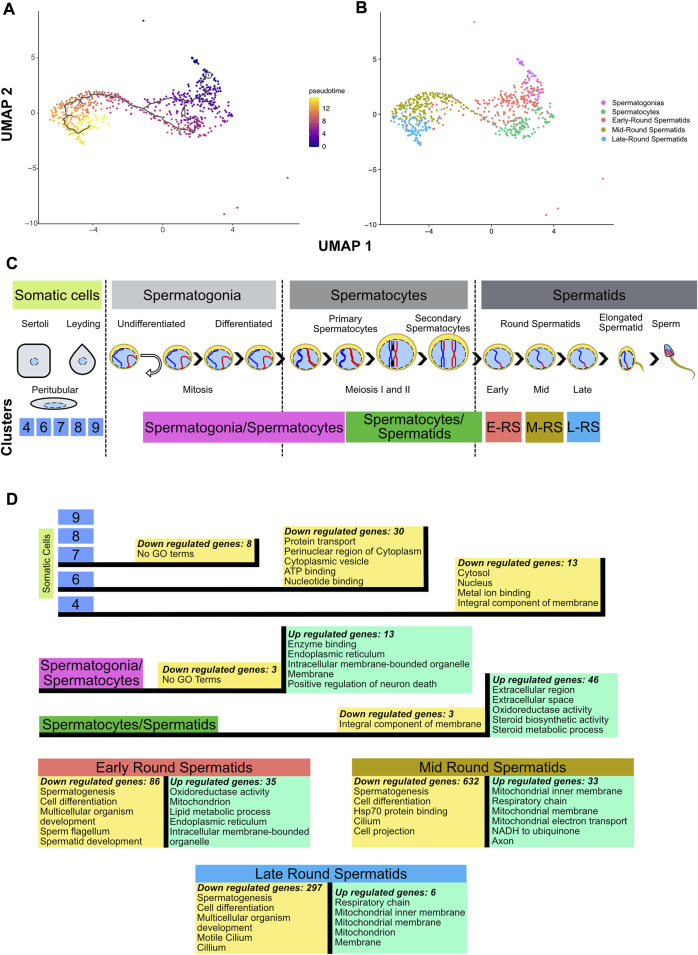
Inferring cell trajectory and functional annotation. **(A)** Pseudotime analysis in spermatogenic cells. Scale color in pseudotime shows the trajectory in specialization cell process. The projection was performed using UMAP and the parameter k = 3 in monocle3 1.2.6. **(B)** UMAP projection without somatic cells and molecular markers identification of cells populations. **(C)** Line time of spermatogenesis process with location of clusters obtained in this dataset based on similarity heatmap, molecular markers identification and pseudotime analysis. E-RS = Early-Round Spermatids, M-RS = Mid-Round Spermatids, L-RS = Late-Round Spermatids. **(D)** Differential gene expression (*p*-value adjusted <0.05) performed between *Ctcf*-cKO against WT cells in each cluster, and the top 5 GO terms of the upregulated (green boxes) and downregulated genes (yellow boxes) in the different cell clusters.

Since clusters 4, 6, 7, 8 and 9 contain somatic cells and cluster 4 also contains type A undifferentiated spermatogonia where the conditional deletion of *Ctcf* is not active (Hernández-Hernández et al., 2016), we decided to remove them from our next analysis (Supplementary Figure S2A). To corroborate the identities and obtain a finer resolution of the clusters with spermatogenetic cells, we performed a single cell trajectory analysis using monocle 3 (Trapnell et al., 2014). With this pseudotime analysis, cluster 5 was stablished as the initial stage (spermatogonia/spermatocytes) followed by cluster 2 (spermatocytes/round spermatids), then cluster 0, cluster 1 and cluster 3. Therefore, these last three clusters, initially identified as round spermatids, can be further identified as early, mid, and late round spermatids (Figures 2A, B). Taken together, we have cell clusters that recapitulate spermatogenesis in both genotypes (Figures 1C, D), which allows the analysis of transcriptional defects in the absence of CTCF in the *Ctcf-*cKO mouse across the last stages of spermatogenesis (Figure 2C).

### Alterations in the transcriptional profiles of spermatogonia and spermatocyte cells

To evaluate whether loss of *Ctcf* produces transcriptional alterations throughout the spermatogenic process. We performed DGE between cells of the same cluster but opposite genotype (*Ctcf-*cKO vs. WT). Then, to obtain a global overview of the biological functions of the proteins encoded by down and upregulated genes, we performed DAVID analysis (with the whole genome of *M. musculus* as background) and selected the top five GO terms with the highest enrichment scores (Huang et al., 2009a; Huang et al., 2009b) (Supplementary Table S1). As expected by the conditional mutation taking place mostly at primary spermatocytes (Hernández-Hernández et al., 2016), we found minor transcriptional alterations between *Ctcf*-cKO and WT cells in somatic cells. While in clusters 9 and 8 we did not find any misregulated genes, in clusters 7, 6 and 4 we found 8, 30 and 13 downregulated genes, respectively (logfc.threshold = 0.25 and *p*—value adjusted <0.05) (see Supplementary Table S1). Most of these genes have GO terms related to signaling process (Supplementary Table S1) except for cluster 7 in which we did not find related GO terms (Figure 2D).

In spermatogonia/spermatocytes cluster, we found 3 down and 13 upregulated genes in *Ctcf-*cKO cells (*p*-value adjusted <0.05). Although downregulated genes did not have associated GO terms, upregulated genes show mostly annotations of cellular components (Figure 2D and Supplementary Table S1), suggesting that transcriptional alterations in spermatogonia and spermatocytes do not produce major changes in the viability and function of these cells. In fact, RNA levels of crucial premeiotic and meiotic markers in these clusters did not show significant differences between *Ctcf*-cKO and WT mice (Supplementary Figure S3). This is in line with our previous data showing that checkpoint-triggering biological pathways (i.e., synaptonemal complex formation and meiotic recombination) are not altered in spermatocytes (Hernández-Hernández et al., 2016).

In *Ctcf-*cKO cells from the cluster of spermatocytes/spermatids, we found 3 down and 46 upregulated genes (*p*-value adjusted <0.05). Downregulated genes are associated with integral components of the membrane, whereas upregulated genes are mainly involved in extracellular and metabolic processes (Figure 2D and Supplementary Table S1).

### Defects in transcriptional profiles in round spermatids lead to abnormal morphology and physiological functions

During spermiogenesis, deep changes in the morphology and physiology of haploid cells produces mature sperm (Torres-Flores and Hernández-Hernández, 2020). Concomitantly, major regulation and changes in transcriptional profiles in round spermatids produce several cell clusters in scRNA-seq datasets (Hermann et al., 2018; Lukassen et al., 2018). Clusters representing early, mid, and late spermatids of *Ctcf*-cKO mice, have fewer upregulated genes compared to downregulated genes. In early round spermatids, the 35 upregulated genes are involved in metabolism activity, while in mid round spermatids the 33 upregulated genes are related to mitochondrial process and axon. Finally, in late round spermatids, the six upregulated genes are related to mitochondrial components (Figure 2D and Supplementary Table S1). On the contrary, we identified 86, 632 and 297 downregulated genes in early, mid and late round spermatids, respectively. All these genes have annotations related to spermatogenesis, spermatid development, sperm flagellum, and cell differentiation process (Figure 2D and Supplementary Table S1).

Since previously we observed severe morphological alterations in mature sperm of *Ctcf*-cKO mice (Hernández-Hernández et al., 2016), we sought to perform a more robust characterization of the overrepresented altered biological pathways during spermatogenesis that could explain the malformation of mature sperm. We performed pathway enrichment analysis using the GSEA tool (Subramanian et al., 2005) and used Cytoscape software for visualization of enriched pathways (represented as nodes of sizes that are proportional to the number of mis regulated genes in the specific gene set). When several pathways are associated by similarity, they are shown as interconnected within circles, representing higher-level processes given a specific biological function (Shannon et al., 2003).

**FIGURE 3:**
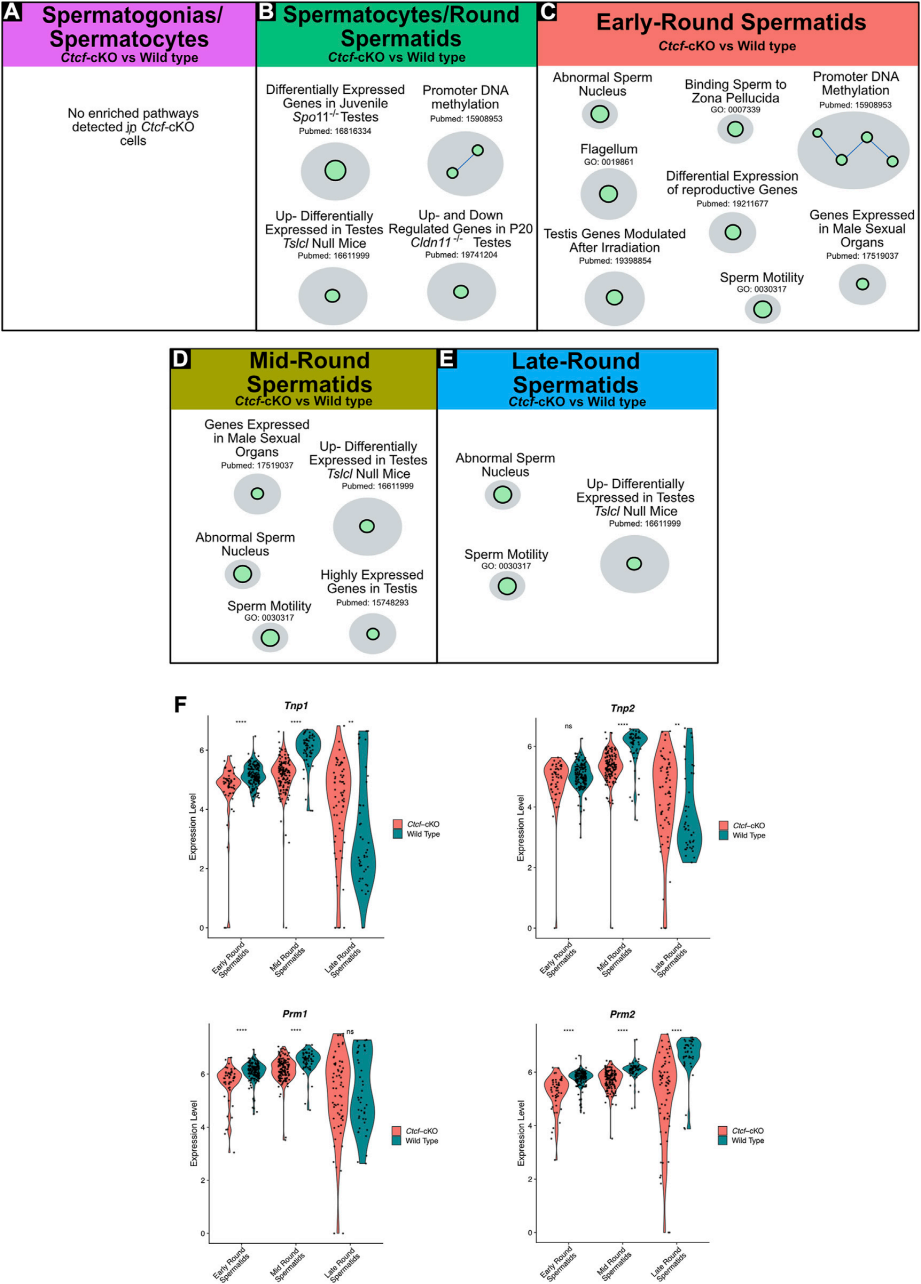
Gene Set Enrichment Analysis (GSEA) of *Ctcf*-cKO against WT spermatogenesis cells and differential gene expression of key players in histones replacement. **(A–E)** Positively enriched maps of altered biological functions in *Ctcf*-cKO *versus* WT cells. The comparisons were made in each cluster. Each green circle (node) represents a pathway. A link between pathways was established when 2 pathways had a Jaccard index >0.4, given the expressed genes. All enriched pathways have an FDR <0.05 and a *p*-value <0.01. **(F)** Violin plots of the expression profiles of *Tnp1, Tnp2, Prm1,* and *Prm2* in round spermatids of *Ctcf-*cKO and WT within cell clusters 3, 2 and 0. Statistical differences are based on the Wilcoxon rank sum test, asterisks indicated *p* < 0.05, expression levels correspond to log normalized values.

With this analysis, we found several biological pathways to be overrepresented (i.e., altered biological pathways) in spermatogenic cells from *Ctcf-*cKO mouse (Figures 3A–E; enrichment scores in Supplementary Table S1). While we did not identify differentially enriched pathways in the spermatogonia/spermatocytes cluster (Figure 3A); in the spermatocytes/spermatids cluster we observed altered biological pathways related to specific gene expression in testis or spermatids, promoter DNA methylation, and differentially expressed genes in KO mice models for spermatocytes/spermatid development (Figure 3B). Among the KO mouse models with similar gene transcriptional defects we found: the spermatid developmental obstructing *Tslc1* null (van der Weyden et al., 2006), the spermatocyte progression stopping *Spo*11^−/−^ and *Cldn*11^−/−^ mice (Smirnova et al., 2006; Mazaud-Guittot et al., 2010).

In early round spermatids we found enriched pathways related to abnormal sperm nucleus, flagellum, sperm motility, and promoter DNA methylation among others (Figure 3C). Finally, in clusters of mid and late round spermatids, we observed physiological pathways involved in sperm motility, abnormal sperm nucleus, and developmental obstructing *Tslc1* null (van der Weyden et al., 2006) (Figures 3D, E). Altogether, our data shows that round spermatids suffer transcriptional alterations that may lead to the mature sperm malformation.

### The protamine incorporation pathway is affected in *Ctcf*-cKO spermatids

To further support our GSEA data, suggesting abnormal spermatid development and defects in the sperm nucleus, we decided to analyze key proteins involved in the histone-to-protamine exchange process (Dadoune, 2003; Torres-Flores and Hernández-Hernández, 2020). First, we observed transcriptional misregulation of *Tnp1, Tnp2, Prm1, and Prm2* in the three stages of spermiogenesis (i.e., early, mid and late round spermatids) (Figure 3F).

**FIGURE 4:**
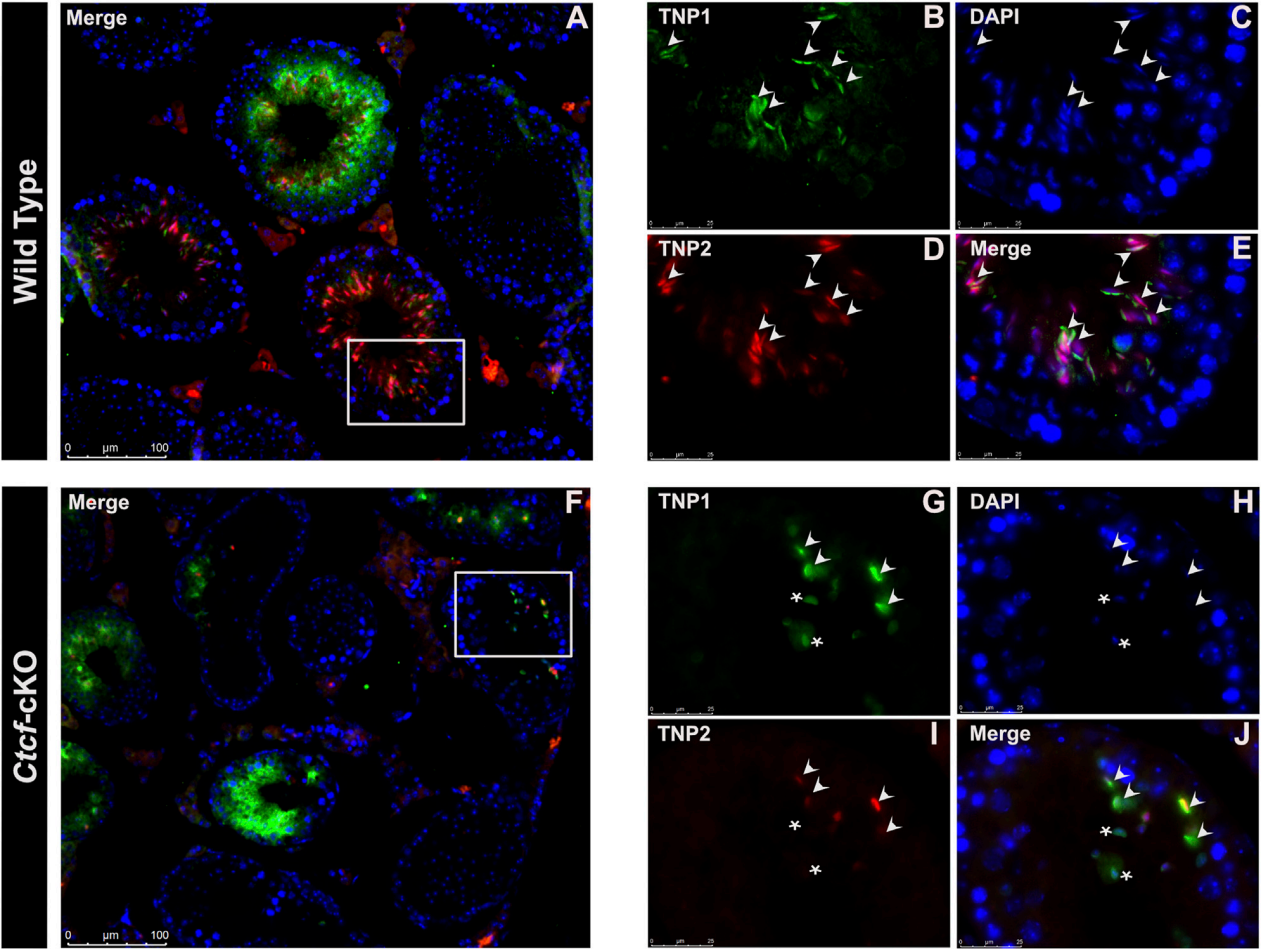
Immunofluorescent staining pattern of TNP1, TNP2, and PRM2 in WT and *Ctcf*-cKO testis sections. **(A–E)** Show simultaneous immunodetection of TNP1 and TNP2 in elongated spermatids from WT testis sections. **(F–J)** Simultaneous immunodetection of TNP1 and TNP2 in elongated spermatids from *Ctcf*-cKO testis sections. Elongated spermatids with immunodetection of both proteins are shown with arrowheads, while abnormal spermatids with signal only for TNP1 are shown with asterisks. 25% of the analyzed seminiferous tubules showed spermatids with an abnormal staining pattern. Three mice of each genotype were analyzed.

Then we decided to follow these proteins at the time of their incorporation in the nuclei of elongating spermatids by immunofluorescence analysis in histological sections of *Ctct*-cKO and WT testes. We observed that whereas in elongated/elongating spermatids from WT mice, both TNP1 and TNP2 proteins are co-expressed (Figures 4A–E), in 25% of the analyzed seminiferous tubules from *Ctcf*-cKO mice some spermatids - without the classical elongated shape - display only TNP1 (Figures 4F–J). Also, to estimate interference by background noise, we run negative controls (histological sections incubated only with secondary antibodies, Supplementary Figure S4).

**FIGURE 5:**
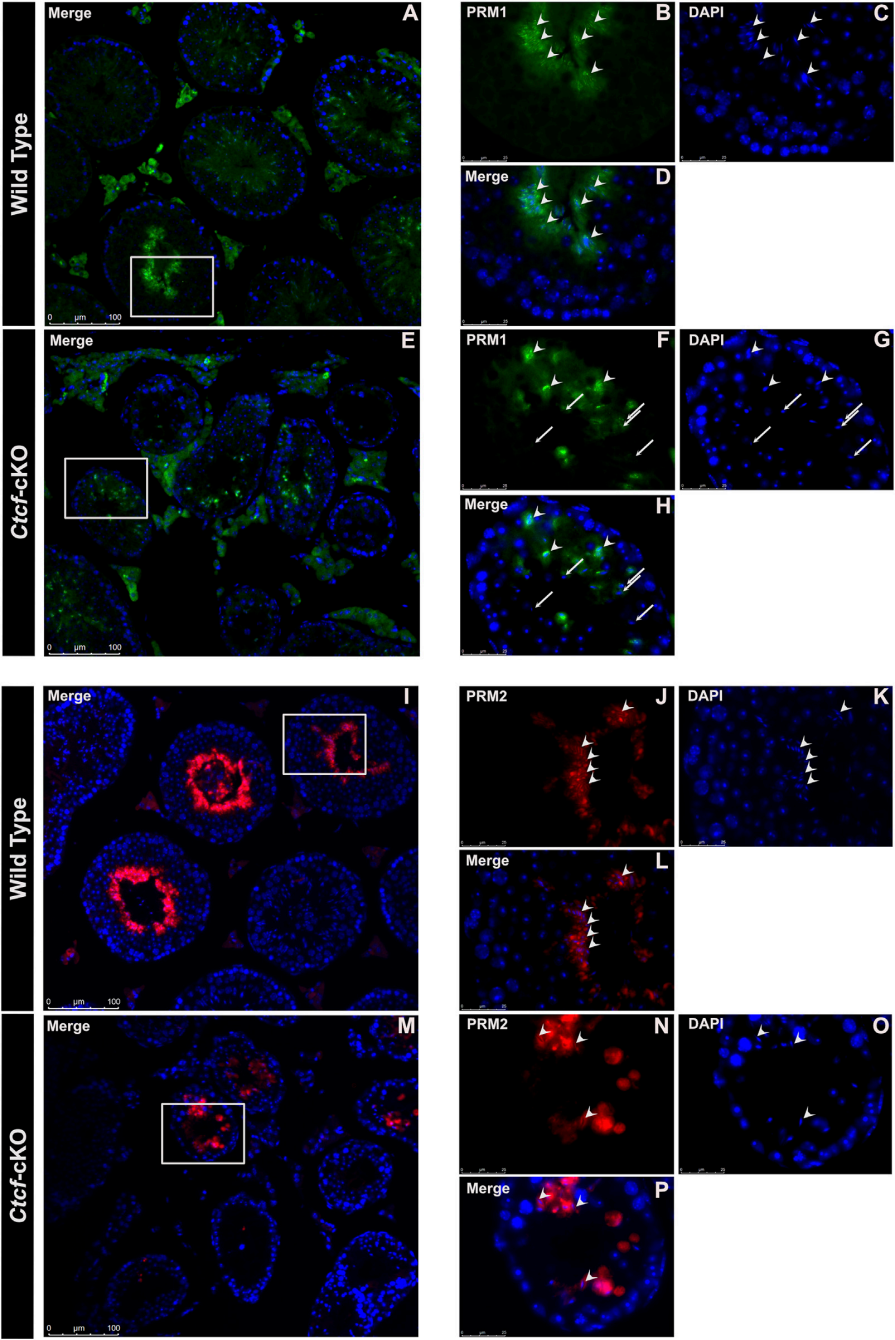
Immunofluorescent staining pattern of PRM1 and PRM2 in WT and *Ctcf*-cKO testes sections. **(A–D)** PRM1 immunodetection in elongated spermatids from WT testes sections. Some representative elongated spermatids with PRM1 signal are pointed out with arrowheads. **(E–H)** Immunodetection of PRM1 in elongated spermatids from *Ctcf*-cKO testis sections. Elongated spermatids with and without the PRM1 signal are pointed out with arrowheads and arrows, respectively. **(I–L)** Immunodetection of PRM2 in elongated spermatids from WT testis sections. **(M–P)** Immunodetection of PRM2 in elongated spermatids from *Ctcf*-cKO testis sections. Elongated spermatids with PRM2 immunodetection are shown with arrowheads.

Elongated spermatids from both WT and *Ctcf*-cKO display PRM2 and PRM1 (Figure 5A–P). However, we found absence of PRM1 signal in some spermatids in all observed seminiferous tubules from *Ctcf-*cKO mice (Figures 5E–H). Furthermore, only 35% and 18% of the analyzed seminiferous tubules from *Ctcf*-cKO displayed signal for PRM1 and PRM2, respectively (Figures 5E–H; Figures 5M–P). Whereas in WT sections, 55.5% and 60% seminiferous tubules displayed PRM1 and PRM2 signal, respectively (Figures 5A–D; Figures 5I–L). Once more, to estimate background noise we use negative controls (Supplementary Figure S5). These data show that the protamine incorporation process is altered in *Ctcf*-cKO spermatids.

### Spermatids in *Ctcf*-cKO mice display abnormalities in acrosome formation and nuclear compaction

Finally, we decided to characterize some of the morphological pathways that are affected in spermatids. Therefore, we performed a detailed morphological characterization of cells in spermiogenesis at the electron microscopy (EM) level. We found mainly two phenotypes in spermatids that support the abnormal sperm morphology reported in the GO and GSEA analysis.

**FIGURE 6:**
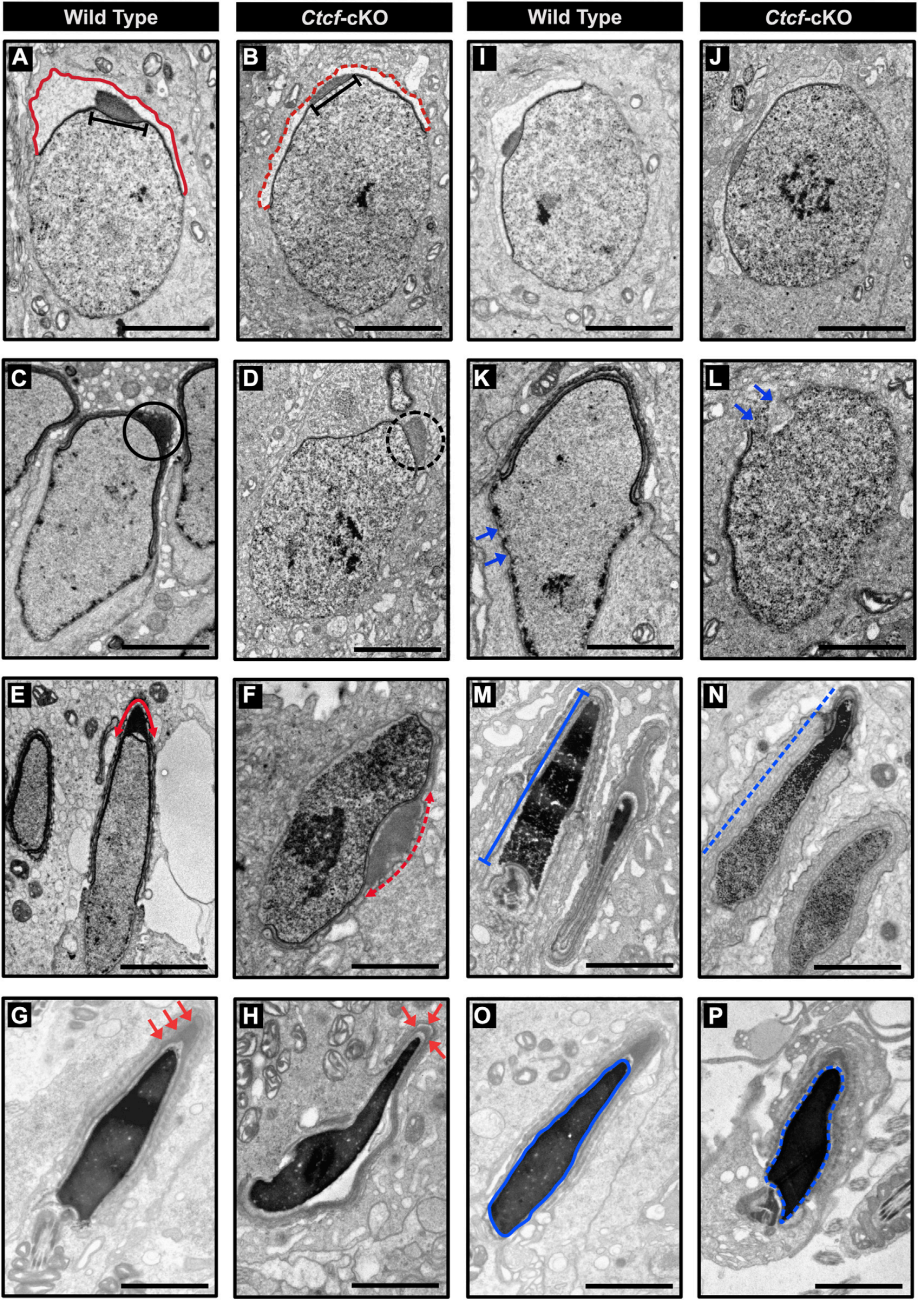
Electron micrographs of spermatids from WT and *Ctcf*-cKO mice. **(A)** Micrograph showing the acrosome sac (red line) and head cap (black bar) of a WT round spermatid. **(B)** Abnormal acrosome sac (red dashed line) and head cap (black bar) of a round *Ctcf-cKO* spermatid. **(C)** Head cap (black circle) from a WT elongating spermatid. **(D)** Abnormal head cap (black dashed circle) of an elongating *Ctcf-cKO* spermatid. **(E)** Acrosome in the anterior apical end of elongated spermatids from WT mice (red double head arrow). **(F)** Misplaced acrosome (red dashed double head arrow) in an elongated spermatid from *Ctcf-*cKO mice. **(G)** Acrosome at the anterior apical end of the elongated spermatids of the WT testes (red arrows). **(H)** Abnormal acrosome and altered morphology in elongated spermatids from testis in *Ctcf*-cKO mice (red arrows). **(I, J)** Proper nuclear morphology and chromatin compaction in round spermatids from WT and *Ctcf*-cKO mice, respectively. **(K)** Elongating spermatid with electron-dense material associated with the nuclear envelope (blue arrows) from WT mice. **(L)** Elongating spermatid with discontinuities in the dense structure associated with the nuclear envelope and in the nuclear envelope itself (blue arrows) from *Ctcf-*cKO mice. **(M)** Elongated spermatid with condensed chromatin (blue bar) from WT mice. **(N)** Elongated spermatid with abnormal chromatin condensation pattern of *Ctcf*-cKO mice (blue dashed line). **(O)** Elongated spermatid displaying full chromatin packing inside the nucleus (blue contoured line) from WT mice. **(P)** Elongated spermatid displaying apparently full chromatin packing (blue contoured dashed line), but with abnormal head morphology from *Ctcf*-cKO mice. Scale bars represent 2 μm.

The first is related to acrosome biogenesis. In round spermatids from WT mice, we observed the acrosome sac and the head cap (Hc) with their expected spherical formation on the surface of the nucleus (Cheng and Mruk, 2010) (Figure 6A), while in round spermatids from *Ctcf*-cKO these two structures are rather flattened (Figure 6B). Following spermatid differentiation from spherical to asymmetric shapes in steps 8–10, we observed that the acrosome and the Hc are condensed and flattened, giving rise to a highly electron-dense acrosome vesicle (Kierszenbaum et al., 2003) (Figure 6C). However, we did not observe these changes in the formation of the acrosome vesicle in *Ctcf-*cKO elongating spermatids (Figure 6D). Furthermore, in elongated spermatids from WT mice, we observed a well-defined acrosome vesicle in the apical region of the elongated nucleus (Figure 6E), while in *Ctcf*-cKO elongated spermatids, we observed a non-well-defined acrosome vesicle in other regions of the nucleus rather than in its apical part (Figure 6F). Finally, we observed that the acrosome in elongated spermatids from WT testis is located at the apical zone as commonly reported (Figure 6G). However, in elongated spermatids from *Ctcf-*cKO testis, the acrosome did not form properly (Figure 6H).

The second phenotype that we underscored was related to nuclear shape (Figures 6I–P). We observed nuclear shape defects in the spermatids of *Ctcf-*cKO mice starting at the stage of elongating spermatids. At this stage, we observed some broken regions of the nuclear envelope in *Ctcf-*cKO elongating spermatids (Figures 6K–L). Furthermore, we observed adequate chromatin compaction in WT elongated spermatids (Figure 6M) but not in elongated spermatids of *Ctcf*-cKO (Figure 6N). Finally, we observed an abnormal nuclear shape in *Ctcf-*cKO elongated spermatids (Figures 6O–P).

## Discussion

Although CTCF is a key molecule for sperm formation, fertility, and sperm epigenome establishment in mice (Jung et al., 2017), little is known regarding its function throughout the different stages of spermatogenesis. In this study, by analyzing the transcriptome of single cells at different stages of spermatogenesis, we found that there are no major alterations in the biological functions of mitotic and meiotic cells under the absence of CTCF. However, haploid cells in spermiogenesis undergo significant changes in their transcriptional profiles and morphology development. Our GO and GSEA analysis of the misregulated genes indicated that early mid and late round spermatids have spermatid development and sperm morphology defects. Indeed, we observed defects in the histone to protamine exchange process, nuclear compaction, and acrosome biogenesis throughout the different stages of spermiogenesis. In a previous work, we reported mature sperm from the cauda epididymis fro*m Ctcf*-cKO mice with acrosome and nuclear compaction malformations (Hernández-Hernández et al., 2016). In this work, we found that these defects are produced by alterations in transcriptional profiles of related genes in round spermatids.

Single-cell RNA-seq studies in mouse spermatogenesis have shed light on the heterogeneity found during this process (Green et al., 2018; Hermann et al., 2018). Since we were interested in identifying changes in the main stages of spermatogenesis due to the absence of CTCF, rather than in deciphering the already reported cell heterogeneity, we performed scRNAseq with fewer cells than in the mentioned studies. Thus, differences in the number of identified clusters may be due to the total number of cells captured in the different studies. However, we used stage-specific markers from the previously reported studies to identify and validate cluster phenotypes in our bioinformatics pipeline. Furthermore, as also observed in similar studies (Lukassen et al., 2018), we observed that some molecular markers are shared across cell clusters which may be explained by the fact that germ cells are embedded into the Sertoli syncytium, thus having structural and signaling interactions (Nishimura and L’Hernault, 2017).

It is well documented that scRNA-seq can underscore cell phenotypes that are otherwise masked when bulk RNA-seq analysis of whole tissues is performed (Bacher and Kendziorski, 2016; Chaudhry et al., 2019). Accordingly, we found that during the round spermatid stage in *Ctcf*-cKO testes, some of the enriched pathways identified in GSEA are related to promoter DNA methylation, a finding that can be further explored in our model since DNA methylation in the germline has the potential to regulate gene expression in the offspring. Hence, failures in this process could have serious consequences for post-fertilization development (Stewart et al., 2016). In mouse, genome-wide demethylation occurs early in the development of primordial germ cells. However, a wave of *de novo* methylation adds epigenetic memory at specific genomic sites (imprinted regions) at the pro-spermatogonia stage before birth (Reik et al., 2001; McSwiggin and O’Doherty, 2018). In our GSEA analysis, we discovered that a pathway related to promoter DNA methylation is affected in *Ctcf*-cKO round spermatids. Although we did not further study this pathway, we speculate that, in a WT scenario, this biological pathway is an ongoing process. Yet, *de novo* DNA methylation during round spermatid development has not been reported. Instead, it has been suggested that active DNA methylation mechanisms, regulated by MORC1, are acting to repress transposable elements in the male germline of mice (Pastor et al., 2014). Accordingly, in our GSEA study we found that MORC1 expression is altered in round spermatids from *Ctcf-*cKO testes.

Although in a small number, sperm with several morphological and biochemical alterations are still produced in the absence of CTCF (Hernández-Hernández et al., 2016). We found most of these defects are created at the transcriptional level in round spermatids, and the resulting phenotype is observed throughout the whole process of sperm differentiation. However, the exact mechanism by which CTCF is controlling these biological functions has not been fully addressed. A possible mechanism may be related to the establishment of the sperm epigenome and histone replacement/retention (Jung et al., 2017), in which CTCF could be deeply involved. At these stages of round spermatids, CTCF and cohesin complexes occupy specific genomic regions, orchestrating the organization of the genome or sperm epigenome (Jung et al., 2017; Jung et al., 2019). It has long been speculated that BORIS (a CTCF paralog) can contribute together with CTCF to organize the spermatid genome. Interestingly, it has been shown that heterodimeric units of CTCF/BORIS can regulate transcription of some round spermatid specific genes, repress toxic genes, and contribute to some extend to the sperm genome organization (Rivero-Hinojosa et al., 2021). Furthermore, ectopic expression of BORIS in BORIS negative cells has been shown to produce the exchange of chromatin bound CTCF homodimers by CTCF/BORIS heterodimers (Pugacheva et al., 2015). Therefore, although the individual contributions of CTCF and BORIS to spermatogenesis are different (Suzuki et al., 2010), it may be interesting to study whether under CTCF depletion of specific genomic locations (in this *Ctcf*-cKO mice), BORIS takes over and binds to these sites. We speculate that the absence of CTCF in round spermatids may have a strong impact on the organization of the sperm epigenome by altering sperm-specific transcriptional regulatory programs. Detailed chromatin accessibility studies at the single cell level are needed to explore this hypothesis. Furthermore, our work highlights the importance of CTCF for the proper progression of spermiogenesis.

## Data Availability

The data presented in the study are deposited in the GEO repository, accession number GSE166774.
