# Supplementary figures and images for: Spermiogenesis alterations in the absence of CTCF revealed by single cell RNA sequencing
Source: Front Cell Dev Biol. 2023 Mar 30;11:1119514. doi: 10.3389/fcell.2023.1119514 (PMC10097911; doi:10.3389/fcell.2023.1119514)

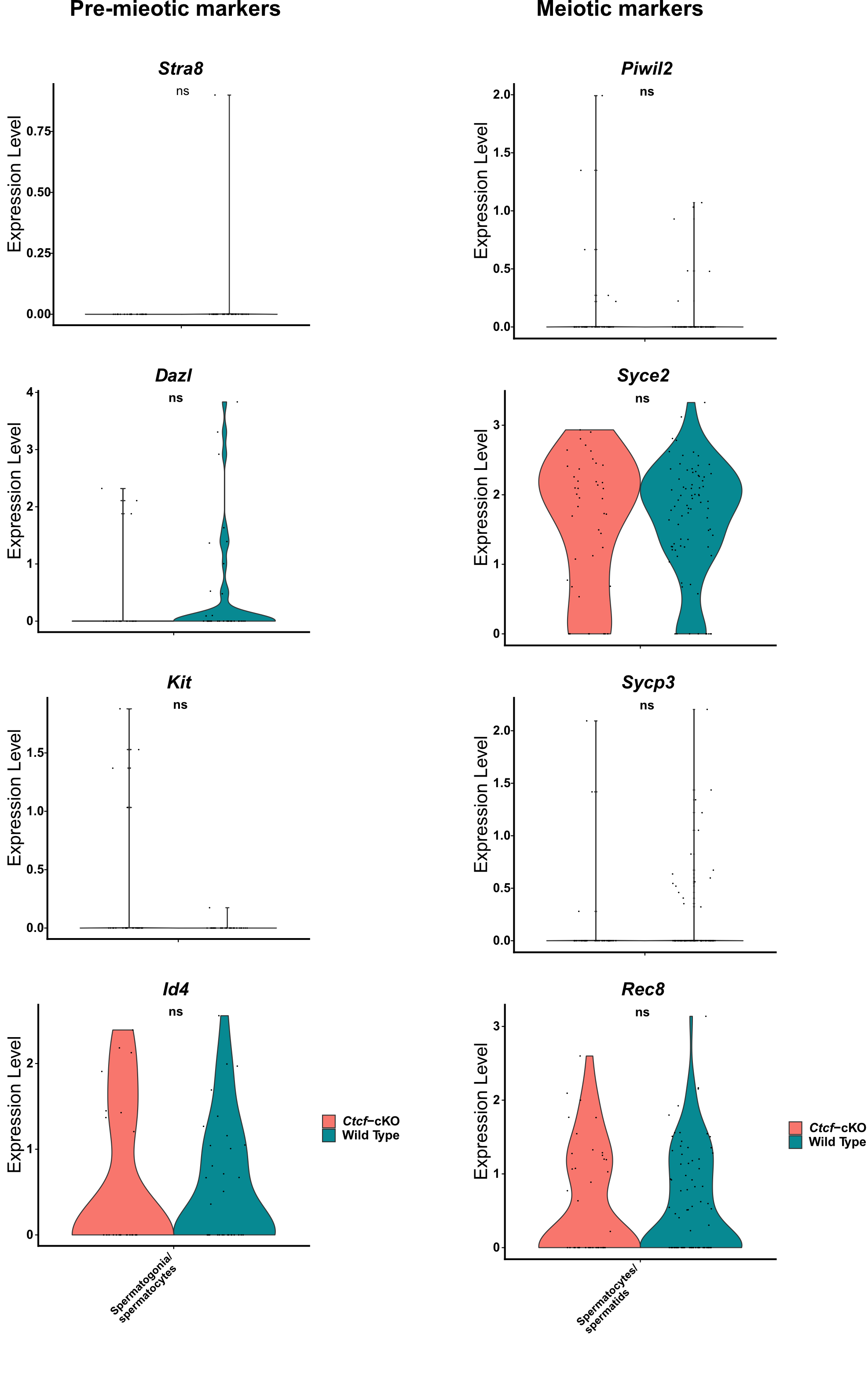

Supplement: Supplementary file 1 [file Image3.TIFF]

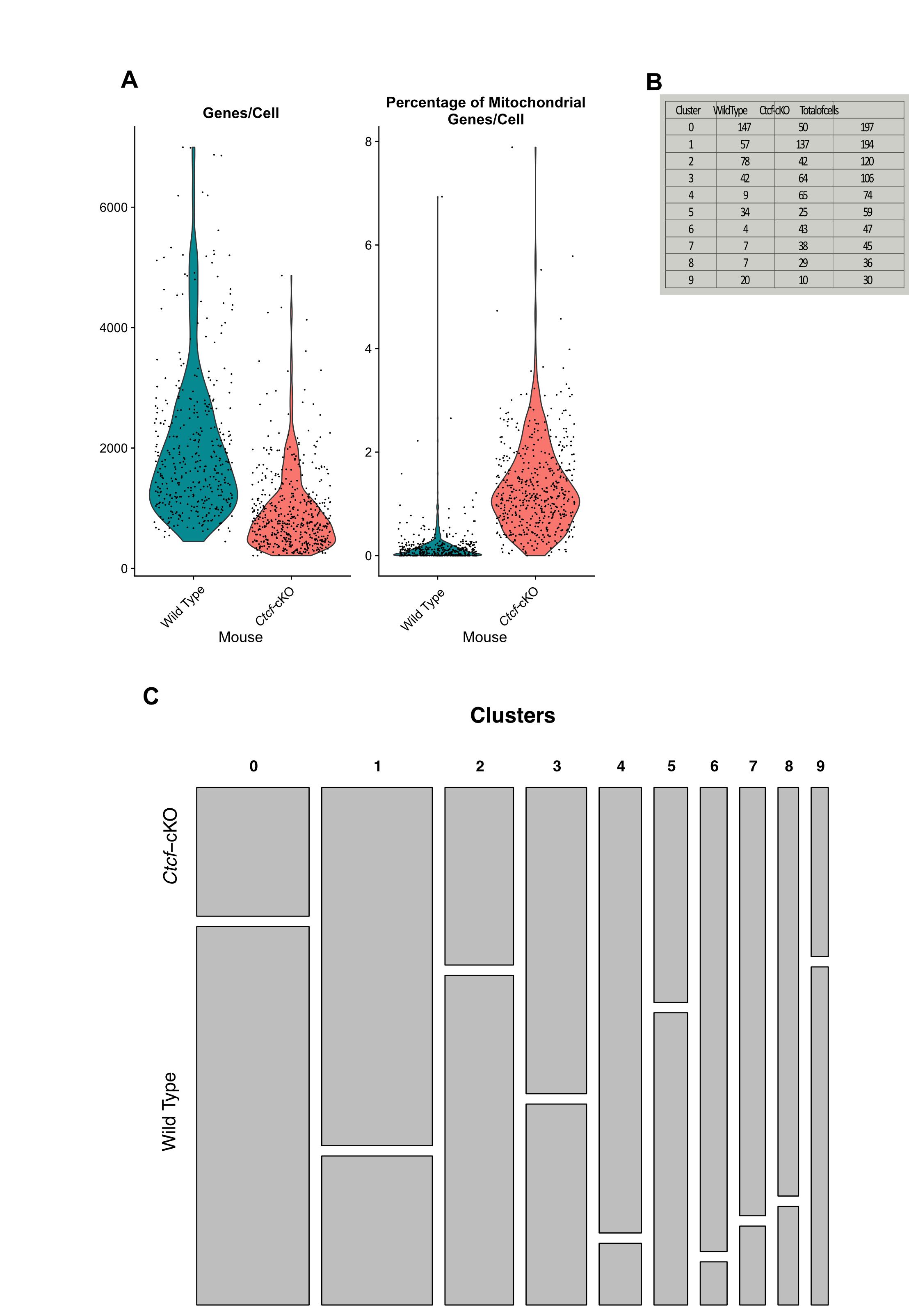

Supplement: Supplementary file 2 [file Image1.TIFF]

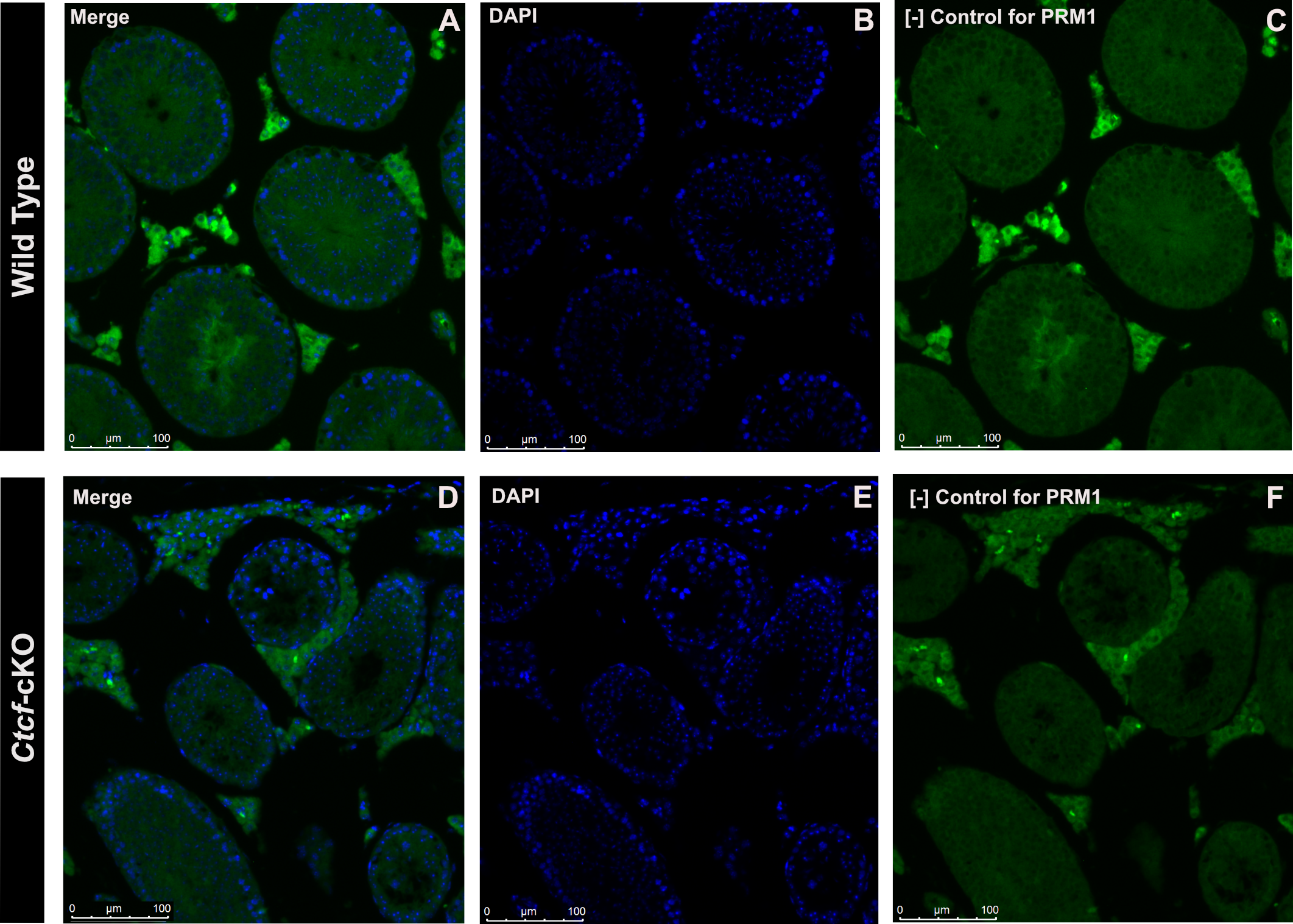

Supplement: Supplementary file 3 [file Image5.TIFF]

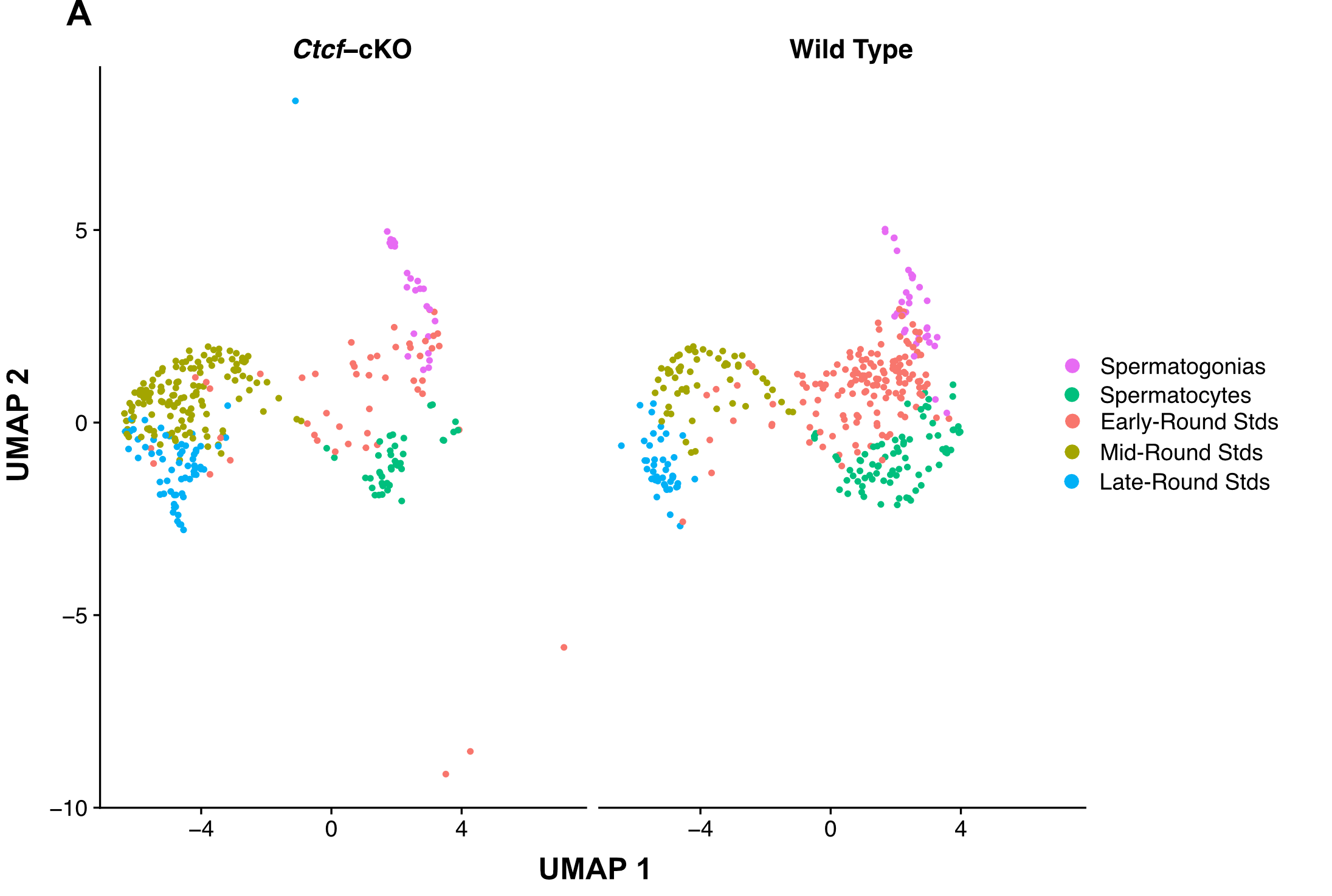

Supplement: Supplementary file 6 [file Image2.TIFF]

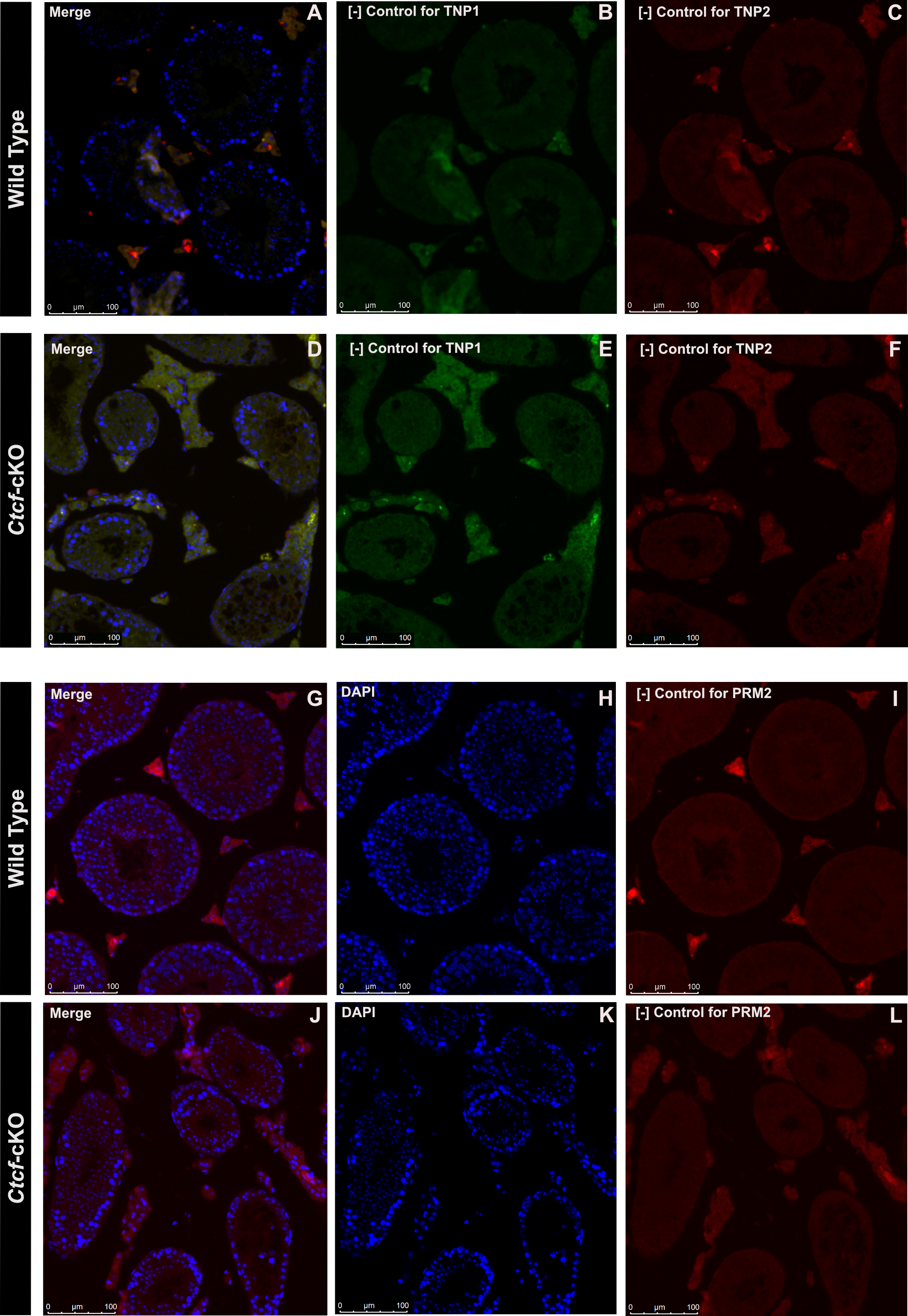

Supplement: Supplementary file 7 [file Image4.TIFF]
