# Supplementary material for: Spermiogenesis alterations in the absence of CTCF revealed by single cell RNA sequencing
Source: Front Cell Dev Biol. 2023 Mar 30;11:1119514. doi: 10.3389/fcell.2023.1119514 (PMC10097911; doi:10.3389/fcell.2023.1119514)
Supplement: Supplementary file 4 [file Table2.DOCX]

Supplementary Material

## Supplementary Figures

**Supplementary Figure S1**. (A) Violin plot of genes and mitochondrial genes per cell in WT and *Ctcf*-cKO testes cells. (B) Table with number of cells that correspond to each cluster from both genotypes. (C) Bar graph with the proportion of cells in each cluster. The thickness of the bar is proportional to the number of cells.

**Supplementary Figure S2.** (A) UMAP visualization of cell clusters by genotype and without somatic cells. Clusters are distinguished by color according to the key color.

**Supplementary Figure S3.** Violin plots displaying the RNA levels of some crucial premeiotic and meiotic genes in cells of the spermatogonia/spermatocytes and spermatocytes/spermatids clusters. Not Statistical differences were identified using Wilcoxon rank sum test, expression level correspond to log normalized values.

**Supplementary Figure S4.** Negative controls for immunostaining. We identified same seminiferous tubules. We used as a negative control, sections incubated with phosphate buffered saline (PBS) instead of primary antibody and using secondary antibody for immunofluorescence detection of TNP1, TNP2 and PRM2.

**Supplementary Figure S5.** Negative controls for immunostaining. We identified same seminiferous tubules. We used as a negative control, sections incubated with phosphate buffered saline (PBS) instead of primary antibody and using secondary antibody for immunofluorescence detection of PRM1.
